# Ultrasound-Guided Injections and Proprioceptive Neuromuscular Facilitation as Shoulder Rehabilitation for Multiple Sclerosis and Neuropathic Pain

**DOI:** 10.3390/healthcare10101869

**Published:** 2022-09-25

**Authors:** Alessandro de Sire, Lucrezia Moggio, Nicola Marotta, Francesco Fortunato, Renata Spalek, Maria Teresa Inzitari, Teresa Paolucci, Antonio Ammendolia

**Affiliations:** 1 Physical Medicine and Rehabilitation Unit, Department of Medical and Surgical Sciences, University of Catanzaro “Magna Graecia”, 88100 Catanzaro, Italy; 2 Institute of Neurology, Department of Medical and Surgical Sciences, University of Catanzaro “Magna Graecia”, 88100 Catanzaro, Italy; 3 Rehabilitation Unit, ‘Mons. L. Novarese’ Hospital, 13040 Vercelli, Italy; 4 Department of Geriatrics, Neurosciences, Orthopedics, Center for Geriatric Medicine (CEMI), Institute of Internal Medicine and Geriatrics, Catholic University of the Sacred Heart, 00168 Rome, Italy; 5 Physical Medicine and Rehabilitation, Department of Oral, Medical and Biotechnological Sciences, Physical Medicine and Rehabilitation, University G. D’Annunzio, 66100 Chieti, Italy

**Keywords:** rehabilitation, multiple sclerosis, shoulder, functioning, upper limb, central neuropathic pain, intra-articular injection, physical therapy

## Abstract

Multiple sclerosis (MS) represents a major cause of chronic neurological disability in young adults and can result in upper limb sensorimotor impairment with a huge impact on manual dexterity and activities of daily living. Moreover, pain is common in MS and a large proportion of patients suffer from central neuropathic pain. To date, no rehabilitative treatment has been described as useful for these patients. A 46-year-old woman, affected by relapsing-remittent MS, described a one-year history of right shoulder pain (Visual Analogue Scale = 8) that started gradually and without trauma. The patient also presented balance and gait impairments, upper limb strength deficit, and fatigue (Expanded Disability Status Scale = 5.5). A multidisciplinary treatment was proposed, including three intra-articular corticosteroid injections and one month of manual therapy, three sessions/week, based on proprioceptive neuromuscular facilitation for the upper limb. At the end of the rehabilitative treatment, pain relief and an improvement in the range of motion of the affected shoulder, upper limb muscle strength, and hand dexterity were observed. The present paradigmatic case report with literature review demonstrated that a multidisciplinary approach seems to be effective in pain relief in a patient with central neuropathic shoulder pain and relapsing-remitting MS.

## 1. Introduction

Multiple sclerosis (MS) is an autoimmune inflammatory demyelinating disease of the central nervous system (CNS) that represents a major cause of chronic neurological disability in young and middle-aged adults [1], affecting approximately 1.3 million people worldwide, with a global prevalence rate of 30 per 100,000 and a female to male ratio of 3:1 [2]. MS is characterized by a wide variety of progressive sensory deficits, reduction in muscle strength, movement coordination, and cognitive and autonomic functions, due to demyelination and axonal loss, influencing the health-related quality of life (HRQoL) of the affected subjects [3]. The patterns of MS presentation are characterized by relapses and/or disease progression: the “relapsing-remitting (RR)” MS, the most frequent one (80% of cases), is characterized by exacerbations and remission [4]. Specifically, patients diagnosed with MS commonly show sensorimotor impairment of lower limbs in 75% of cases and upper limbs in 66% [5]. Furthermore, considering that arm and hand functioning level is greatly related to independence in activities of daily living (ADL) such as eating, dressing, and grooming, 76% of people with MS experience problems with manual dexterity, and 44% experience deficits in ADL [6]. To date, no pharmacological approaches are available to cure MS, but corticosteroid administrations might prevent and treat relapses [7], and immunomodulatory and immunosuppressive therapies could modify the disease course [8,9]. Symptomatic treatments are also necessary to reduce disabling conditions such as spasticity, pain, depression, and bladder dysfunction [10].

Currently, even though several studies have evaluated rehabilitation approaches’ role in MS [2,3,11,12,13,14], there is still a lack of evidence, especially with respect to the treatment of musculoskeletal pain in patients with MS. Multidisciplinary rehabilitation programs, including physiotherapy, occupational therapy, strengthening exercises, endurance training, stretching, orthotics and casting, transcutaneous electric nerve stimulation, hippotherapy, vibration therapy, psychological interventions, nutritional interventions, and specific rehabilitation approaches (such as telerehabilitation, fatigue management, upper limb rehabilitation, and spasticity management) are essentials to improve functional independence and enhance participation [15].

Shoulder pain is a common musculoskeletal symptom in the adult population, representing the third most common symptom presenting to primary care physicians [16] and affecting between 7 and 26% of adults [17]. The diagnosis is usually based on the patient’s clinical condition and careful history taking, without a reliable classification system [18]. Rotator cuff pathology accounts for >60% of shoulder pain and subacromial impingement syndrome is a multifactorial associated diagnosis that is often the result of kinesthetic dysfunction [19]. Physiologically, during activities that involve humeral movement, the rotator cuff muscles maintain the humeral head a few millimeters of the center of the glenoid fossa; in the case of dyskinetic movements, the humeral head moves superiorly resulting in an impingement between the anterior or lateral edge of the acromion and the humeral head, with a high risk of rotator cuff lesions [20,21]. Shoulder impingement syndrome may result from rotator cuff tendonitis or shoulder bursa inflammation, known as subacromial bursitis, which includes pain in passive abduction [22]. This pathological condition results in rotator cuff tendons crushing between the coracoacromial arch and the humeral head [23,24].

Pain, considered as either nociceptive or neuropathic, is one of the most common associated and treated symptoms in MS, estimated to comprise about 30% of all symptomatic treatments [25]. Pain in MS was also widely classified according to its phenomenology and underlying pathophysiological mechanisms into continuous central neuropathic pain, intermittent central neuropathic pain, musculoskeletal pain, and mixed neuropathic and non-neuropathic pain [26,27]. Osterberg et al. indicated that pain might be considered as common in MS with a large proportion of MS patients suffering from central neuropathic pain (at least one third) [28]. Shayesteh Azar and colleagues reported a 34.8 percentage of shoulder pain in a cohort of 115 MS patients, with a significantly higher prevalence in the female population [29].

Although several conservative pharmacological treatments are commonly administered in clinical practice, there is a gap in the literature regarding their efficacy [30]. One of the most generally applied interventions for shoulder pain treatment, regardless of its etiology, is ultrasound-guided corticosteroid injections into the glenohumeral joint through an anterior or posterior approach [31,32]. However, it has been shown that the benefits of corticosteroid injections are unpredictable and short term [33,34,35]. The rehabilitation treatment aims to restore the physiological range of motion through the correct kinematics of the glenohumeral and scapulothoracic joints and is therefore essential for reducing pain and improving function, with similarly positive short- and long-term clinical outcomes as non-conservative approaches [36,37]. Thus, rehabilitation plays a fundamental role in handling the complications of multiple sclerosis and shoulder pain. Furthermore, although the MS pain mechanisms are still poorly understood, 66% of patients develop pain-related upper limb motor impairments, and the prevalence of pain in the shoulders is higher in females compared with males [29], dramatically affecting many daily living activities [38,39]. Its management represents a hard challenge for physicians: pharmacological agents, such as anticonvulsants, non-steroidal anti-inflammatory drugs, antidepressants, and opioids, show several side effects and less than 60% of patients obtain even partial relief [40]. Moreover, the rehabilitation treatment also acts on other features of MS, such as spasticity, sensory-motor alterations, muscle strength, and range of motion, with an increase in the dexterity of the involved limb [41]. It is also important to underline how the functional recovery of the shoulder joint complex is essential to improve the functioning of the entire upper limb and, consequently, HRQoL and disability [19,30,42]. Then, considering these premises, the aim of this paradigmatic case report and a literature review is to evaluate the impact of a conservative approach, including intra-articular corticosteroid injections and physiotherapy, in a woman affected by shoulder pain suffering from MS, in terms of pain reduction, improvement of balance, and consequent improvement in HRQoL.

## 2. Case Presentation

A 46-year-old woman, affected by RR MS since 2001 with an EDSS score of 5.5 referred to the Rehabilitation Unit of the University Hospital “Mater Domini” of Catanzaro, Italy in April 2021, presenting with an upper limb pain and an impairment of physical functioning. The patient did not present all her previous documentation, but she was reported to be under treatment with Tysabri^®^ (natalizumab) i.v., under the supervision of a neurologist; she reported that she was previously treated with interferon, which was discontinued due to adverse effects (flu-like syndrome). Furthermore, the patient immediately showed a weak acceptance of her disabling condition, expressing the desire not to be treated for the neurological disease she was suffering from. The patient, who works as a street vendor at the market with her husband, reported being in spontaneous menopause for two years, without replacement treatments. She described a one-year history of right shoulder pain that started gradually and without trauma, treated with oral anti-inflammatory agent and analgesic, such as non-steroidal anti-inflammatory drugs, with low clinical benefits. In the past year, although she did not undertake brain and spinal cord magnetic imaging (MRI), she did not report exacerbations of MS and did not assume cortisone treatments. Her symptoms worsened in the prior three months due to a chronic painful right shoulder exacerbation, related to an inveterate subacromial impingement syndrome. The patient previously underwent imaging investigations: the shoulder bilateral X-rays showed minimal superficial erosions affecting the lesser humeral tuberculum and greater humeral tuberculum and ipsilateral acromion-clavicular arthrosis, and the ultrasound revealed insertional tendinopathy of the right rotator cuff with fluid accumulation in the subacromial bursa, due to an inflammatory process. During the week before the clinical visit, the patient reported a burning and electric shock sensation in the right upper limb, associated with numbness in the same site. In addition, tactile hypoesthesia and itching of the volar portion of the arm and forearm was detected at the clinical evaluation. Due to this, a DN4 questionnaire, a validated clinician-administered screening tool for neuropathic pain detection, was administered to the patient, resulting in a score of 5, suggesting a neuropathic pain condition. According to Finnerup et al. [43], the diagnosis of neuropathic pain was suspected in our patient for the presence of suggestive, albeit not pathognomonic symptoms, such as burning and electric shock sensation in the right upper limb, associated with numbness in the same site, not responsive to treatment with NSAIDs. Then, unspecified antiepileptic drugs were administered, with temporary clinical benefits and disabled adverse effects, such as asthenia and nausea, which led to poor compliance and subsequent suspension. Furthermore, the same adverse effects were reported following the administration of cannabinoids (Sativex^®^, GW Pharmaceuticals, Cambridge, United Kingdom).

**Figure 1:**
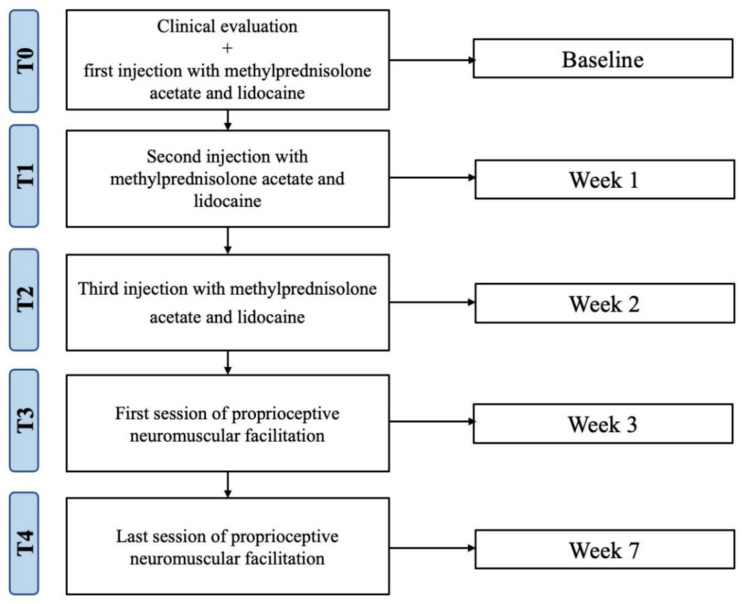
Study flow chart.

The patient presented an independent gait pattern with the need for assistance for moving in external environments, for long distances, and using stairs. She also declared that she needed help in carrying out the ADL, especially regarding the instrumental ADL; however, she reported that she was independent in self-care, with a value of 109 according to the Functional Independence Measure. At the clinical evaluation by a physician specialist in physical medicine and rehabilitation, the patient showed balance and gait impairments, upper limb strength deficit, and fatigue; moreover, she reported a rest pain value equal to 5 on the Visual Analogue Scale (VAS). At the clinical evaluation of the right shoulder, the patient reported acupressure pain on the anterolateral and posterior acromion edge, which worsened with any limb lifting activity. During the passive mobilization, a “click” sound and a “crackling” sensation could be heard. The provocative tests (Neer test and Hawkins test) were positive, with an algo-functional limitation mostly in passive internal rotation and abduction, due to moderate pain. During the passive movements, the patient reported a pain equal to 8 on the VAS, resulting in a functional limitation in all the active shoulder movements. During the clinical evaluation, the following tests were performed to exclude a glenohumeral instability: anterior apprehension test, anterior load and shift test, Drawer test, relocation test, and anterior release test, with negative results. Before treatment, the subject carefully read and signed the informed consent and medical privacy form, and the procedures were in accordance with the Declaration of Helsinki. The case report flow chart is depicted in Figure 1.

**Figure 2:**
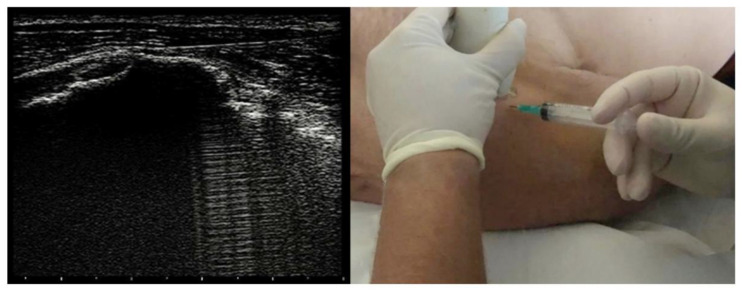
Ultrasound-guided right subacromial bursa injection via anterior approach.

The treatment consisted of three injections of corticosteroid and anesthetic into the subacromial bursa, once a week for three weeks; at the end of this intervention, a rehabilitation treatment focused on shoulder function impairments and gait and balance deficit. Intra-bursal injections were ultrasound-guided via anterior approach with methylprednisolone acetate 40 mg/mL fl 1mL and 2 mL of lidocaine 20 mg/mL and aimed to reduce joint pain at rest and during passive and active mobilization (see Figure 2).

**Figure 3:**
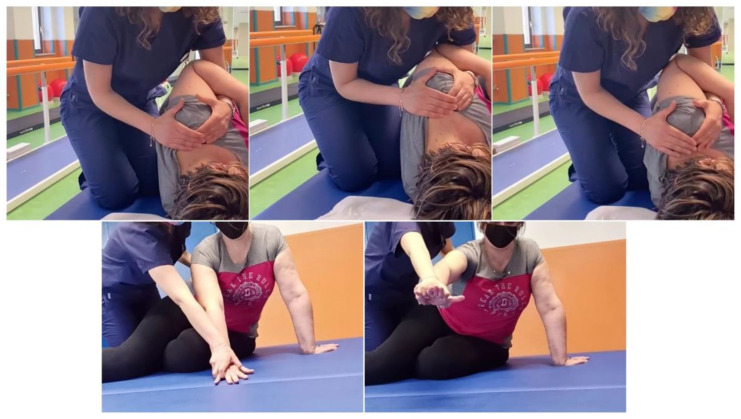
One-month rehabilitation based on proprioceptive neuromuscular facilitation.

After this intervention, we planned a one-month rehabilitation protocol, comprising three sessions/week for four weeks. Each session lasted one hour and was based on Kabat proprioceptive neuromuscular facilitation (PNF) [41] for the upper limb, as depicted in Figure 3. PNF is a widely used rehabilitative approach in clinical practice that might help patients in achieving the highest function level, through a body proprioceptive system, in order to facilitate or to inhibit muscle contraction [44,45,46]. Indeed, the regulation of muscle contraction might be obtained with a stimulus-response model through a feedback motor system [47,48]. The rhythmic initiation consists of scapular complex movement stimulation, with a progression of initial passive, active-assistive, and active movement through the agonist pattern. In particular, the patient was asked to flex her shoulder anteriorly and to depress it posteriorly towards the spine; when she was able to perform both movements, she was asked to mutually activate anterior elevation and posterior depression. The exercise was performed symmetrically both on the painful shoulder and on the contralateral for a total of ten repetitions on each side [49]. Each session opened with a rhythmic initiation with a consequent learning of the movement pattern, with subsequent muscle lengthening and recruiting. The difficulty and the number of repetitions increased according to the patient’s compliance, through the resistance offered by the physiotherapist to movement and with the maintenance of progressively more complex postures. The following exercises focused on shoulder muscle lengthening and recruiting, and on trunk stability improvement, employing the contraction-held-relaxation-elongation technique, in which the patient was asked for a short concentric isotonic contraction followed by isometric contraction of the antagonist muscle, and the holding-relaxation-elongation one, in which only the isometric contraction of the antagonist was requested. The contraction was maintained for 10 s (reaching up to 20 s in the strongest muscles) [50]. The movements were realized according to the facilitation model of the diagonal patterns: for the upper limb, it included shoulder flexion, abduction, and external rotation, encompassing elbow, wrist, and fingers [51]. Moreover, during the treatment sessions, exercises for improving the gait cycle using the Sensory Treadmill (Gait Trainer Treadmill Biodex, BTS Bioengineering Spa, Garbagnate M.se, Milano, Italy) and balance exercises, using a stabilometric platform with a visual cue, to correct the forward displacement of the body’s center of gravity, were performed. The patient completed the rehabilitation program without any interruption for the entire study protocol and without any side effects.

To evaluate the effectiveness of the rehabilitation protocol on our RR MS patient suffering from chronic shoulder pain, we assessed the following outcome measures:

(i) pain, assessed by VAS;
(ii) passive range of motion (ROM) in flexion, extension, abduction, adduction, internal and external rotation, and muscle strength, measured by the Medical Resource Council (MRC) Scale of shoulder flexors, extensors, abductors, adductors, and internal and external rotators;
(iii) upper limb muscle strength, using the Hand Grip Strength Test (HGS), to measure the maximum isometric force exerted by the muscles of the upper limb through a dynamometer [52,53];
(iv) hand dexterity, through the Nine-Hole Peg Test (NHPT), the most used measure in the literature and clinical practice, which consists in evaluating the time needed to insert and then remove, one at a time and as quickly as possible, nine pegs in as many holes on a tablet [54];
(v) upper limb functioning, using the Quick Disability of the Arm, Shoulder and Hand (Quick DASH), including eleven questions regarding upper limb functionality and pain. The score obtained (from 0 to 100) indicates the degree of disability (0 = absence of disability; 100 = maximum disability) [55];
(vi) Berg Balance Scale, comprising 14 tests, each rated from 0 to 4; the sum of the scores indicates the balance level [56];
(vii) Timed Up and Go Test (TUG), to assess the risk of falling. The patient, starting from the sitting position, is instructed to get up on the therapist’s instructions, to walk three meters, to turn on himself or around an obstacle, return to the chair, and sit down [57];
(viii) Ten Meter Walk Test (10MWT), to evaluate the walking speed in meters per second over ten meters [58];
(ix) Fatigue Severity Scale (FSS), a one-dimensional nine-item questionnaire that collects information on the severity and impact on the quality of life of MS fatigue through a seven-point Likert-type scale [59];
(x) European Quality of life—five dimensions—three levels (EQ5D3L) index and EQVAS, a self-administered questionnaire consisting of two different parts. The first one explores five dimensions of interest, such as mobility, personal hygiene, social activities, pain, and anxiety/depression; every single dimension provides three levels of severity (no problem, problem of some entity, problem of extreme gravity). The second section is composed of a 20 cm VAS scale on which the patient indicates the best (score = 0) or the worst (score = 100) possible perceived health status [41].

All the above-mentioned outcomes were assessed at baseline, before the first injection (T0); one week after, before the second injection (T1); after two weeks, before the third injection (T2); after three weeks, before the rehabilitation treatment (T3); after seven weeks, at the end of rehabilitation treatment (T4). All data were recorded, classified, and analyzed by Graphpad Prism 7.0 (GraphPad Software, Inc., San Diego, CA, USA). Descriptive statistics were used to analyze the data. Results are presented as the mean and standard deviation for continuous variables and counts and percentages for dichotomous, nominal, and ordinal variables.

At the end of the multidisciplinary treatment, we identified an improvement in all the outcome measures. The VAS identified a reduction in pain already, following the three injections. Regarding the instrumental evaluation, the patient achieved a score of 15 at T0, 17 at the end of the infiltration cycle at T3, and 20 at the end of the treatment sessions at the HGS of the affected limb.

The value obtained at the TUG was 25.41 at T0, not significantly reduced after the last injection, but decreasing by about 8 s at T4. The 10MWT recorded a score of 20 s at T0 recovering one second at the end of the infiltrative cycle and decreasing at 17 s at T4.

Regarding the active ROM of the right shoulder and the strength assessed by the MRC scale, the patient showed a significant improvement in all planes and movements between T0 and T4. The ROM values are shown in Table 1.

## 3. Discussion

The aim of the present case report, with a literature review, was to evaluate the impact of multidisciplinary rehabilitation treatment in pain reduction and improvements in HRQoL in a woman with central painful shoulder suffering from MS. Thus, we aimed to provide a literature review on rehabilitation treatment in this type of patient. The treatment resulted in a five-point decrease in VAS. This achievement allowed for greater compliance with the treatment, with the consequent recovery of the ROM of the shoulder, an increase in the functionality of the upper limb and its inclusion in the ADL, and an improvement in the patient’s quality of life. Moreover, although MS non-stable disease course RR MS is considered as a risk factor for greater pain severity in MS patients [60], our case highly suggests a multidisciplinary approach of MS pain-related comorbidities even in patients with apparently clinically stable disease. The case reported in this paper is paradigmatic as the patient presented, in addition to the condition of disability resulting from the MS disease from which she suffered since 2001, a central painful right shoulder with a significant impact on HRQoL. Although the effects of the multidisciplinary rehabilitative approach appear to be more effective in MS patients with a “mild” EDSS score [61], we also observed a significant beneficial effect in outcome measures even in an MS patient with a “moderate-severe” EDSS score. Although the approach employed for the treatment of this patient is mainly involved in the rehabilitation of MS, its application to the shoulder joint complex, through the phenomenon of irradiation, allowed a general involvement of the upper limbs and trunk, with a consequent improvement in trunk control and hand functioning. Moreover, the PNF application in the treatment of a painful shoulder also finds evidence in the scientific literature. In 2020, Peteraitis et al. [62] aimed to prove the feasibility of the PNF technique administration in a patient suffering from subacromial conflict syndrome, not responsive to standard physiotherapy. The five-week rehabilitation protocol proposed in this case report allowed the achievement of improvements in pain and ROM. Furthermore, this paper also enables the comparison of PNF with other standard physiotherapy methods, in relation to the failure of previous therapies. A recent randomized controlled trial [50] compared the short-term effects of scapular PNF techniques and classical physiotherapy interventions on pain, scapular dyskinesia, ROM, and joint function in patients with adhesive capsulitis. The 53 subjects were assigned to three groups: the first group received scapular PNF exercises and instrumental physical therapy, such as transcutaneous electrical nerve stimulation (TENS) and ultrasound therapy; the second group underwent standard physiotherapy and instrumental physical therapy; the third group performed only instrumental physical therapy. The authors concluded that both PNF scapular exercises and classical exercise approaches combined with instrumental physical therapy were effective for short-term improvement of shoulder joint functioning. İğrek and Çolak [63], in a recent RCT, compared the effectiveness of PNF and shoulder mobilization in addition to conventional physiotherapy on pain, ROM, functionality, and muscle strength in patients with subacromial impingement syndrome, and concluded that it is recommended that PNF or shoulder mobilization is added to conventional treatment for four weeks. Al Dajah [64] analyzed the PNF effect on 30 patients with painful and limited glenohumeral ROM activities and found that soft tissue mobilization for the subscapularis for 7 min and five repetitions of PNF technique followed by five repetitions of a PNF-facilitated abduction and external rotation diagonal pattern was effective in reducing pain and improving glenohumeral external rotation and overhead reach during a single intervention session. In 2019, a systematic review and network meta-analysis [65] investigated the effects of PNF stretching exercise and kinesiotaping in adults affected by shortness of the pectoralis minor, a potential mechanism underlying shoulder impingement syndrome. The authors affirmed that, compared with kinesiotaping alone and no intervention, PNF stretching exercises increased pectoralis minor length. Çelik et al. [66] compared PNF and myofascial release technique effectiveness in 30 patients suffering from subacromial impingement syndrome on pain, ROM, muscle strength, quality of life, functionality, and disability. After the treatment, shoulder pain, range of motion, muscle strength, functionality, and disability were improved in both the PNF and myofascial release technique groups, but PNF was more effective in reducing activity pain. Moreover, PNF demonstrated a positive impact on poststroke shoulder pain and ROM, and helped in the strengthening of proximal muscles of the upper extremity (UE), thereby correcting scapular alignment, and improving the UE function in stroke patients [67]. However, the scientific literature provides low evidence on PNF efficacy in the treatment of MS patients. In a study published in 2020, Tollár et al. [41] analyzed the effects of five types of rehabilitation treatment on the motor symptoms of 68 MS patients and found that PNF did not improve motor impairments and quality of life more than exergaming, cycling, and balance exercises; however, it should be noted that in the aforementioned study, PNF was applied for the lower limbs, considering balance and gait as outcome measures. Korkmaz at al. [68] compared the effects of high voltage pulsed galvanic stimulation and PNF technique on fatigue and strength in 33 MS patients, finding that PNF was helpful in obtaining more general effects. Olędzka and colleagues [69], in a pilot study, aimed to assess the impact of single-session PNF therapy on the shoulder range of motion and pain level in patients with subacromial impingement syndrome. The experimental group consisted of 11 patients undergoing therapy based on the PNF concept, whereas 12 patients in the control group underwent laser therapy, magnetic field therapy, and local cryotherapy. They concluded that single-session therapy with the use of the techniques and PNF may improve both the active and passive range of shoulder movement, and treatment was positively perceived by patients. In a recent systematic review with meta-analysis [70], Tedla and Sangadala stated that the PNF group was superior in decreasing pain and reducing disability, increasing ROM (especially external rotation and abduction), and improving function. It is necessary to underline that the PNF method does not represent the first choice in the shoulder joint complex function impairment, which often results in participation restriction and activity limitation, but is generally applied in neurological disease rehabilitation. In this paradigmatic case report, the specific treatment of the upper limb joint through PNF led to balance and gait improvement due to the neurological pathology and CNP.

Moreover, Rah et al. reported that subacromial corticosteroid injection showed improvement in hemiplegic shoulder pain (HSP), disability, and active range of motion, and the duration of its efficacy continued up to eight weeks [71]. A recent review and meta-analysis performed by our group confirmed the need to integrate conventional rehabilitation with other rehabilitative techniques that are more effective in reducing HSP [72].

The major findings might confirm the underestimation of patients with MS with chronic and excruciating central neuropathic pain, and the need for a rehabilitative approach aimed at facilitating the degree of pain excitability [26].

This paper is not free from limitations: first, the study design does not allow us to obtain strong conclusions, even though it is a paradigmatic clinical case, and, to the best of our knowledge, the first in the literature; second, the last assessment was at the end of the rehabilitation treatment, not allowing us to analyze any medium- and long-term effects of the PNF technique approach; third, the concomitance of a disabling pathology such as MS in a patient with an exacerbation of pain in a subacromial syndrome might influence and hide the multidisciplinary treatment benefits; lastly, it is absolutely necessary to take into consideration the few neuro-biomechanical studies present in the scientific literature.

## 4. Conclusions

In conclusion, the present paradigmatic case report with literature review demonstrated that a multidisciplinary rehabilitative approach is effective in pain reduction in a patient affected by central neuropathic shoulder pain and suffering from RR MS. Furthermore, the patient also showed joint mobility, muscle strength, upper limb functioning, fatigue, balance, walking, and HRQoL improvement.

Future studies with larger samples and longer follow-ups are still necessary to confirm the effects of this combined treatment in neurological patients starting from a rehabilitation point of view.

## Figures and Tables

**Table 1:** ROM and MRC assessed at the different time-points.

	T0	T1	T2	T3	T4
*Right shoulder ROM*					
Active flexion	60°	60°	70°	90°	180°
Passive flexion	100°	100°	110°	110°	180°
Active extension	30°	30°	30°	30°	40°
Passive extension	30°	35°	35°	35°	45°
Active abduction	75°	80°	85°	85°	180°
Passive abduction	90°	100°	110°	110°	180°
Active adduction	30°	30°	30°	35°	35°
Passive adduction	30°	30°	35°	35°	40°
Active external rotation	45°	50°	50°	55°	70°
Passive external rotation	50°	50°	60°	60°	60°
Active internal rotation	30°	30°	30°	30°	35°
Passive internal rotation	35°	35°	35°	35°	35°
*Right shoulder MRC*					
Flexors	2	3	3	3	4
Extensor	3	3	3	3	4
Adductors	2	3	3	3	4
Abductors	3	3	3	3	4
External rotators	3	3	3	3	4
Internal rotators	3	3	3	3	4

T0: baseline, before the first injection; T1: one week after, before the second injection; T2: after two weeks, before the third injection; T3: after three weeks, before the rehabilitation treatment; T4: after seven weeks, at the end of rehabilitation treatment. Legend: ROM: Range of Motion; MRC: Medical Research Council.

## Data Availability

Dataset is available on request.
